# Simulating the ion permeation and ion selection for a eukaryotic voltage-gated sodium channel Na_V_PaS

**DOI:** 10.1007/s13238-018-0522-y

**Published:** 2018-03-12

**Authors:** Juanrong Zhang, Wenzhi Mao, Yanhui Ren, Rui-Ning Sun, Nieng Yan, Haipeng Gong

**Affiliations:** 1 MOE Key Laboratory of Bioinformatics, School of Life Sciences, Tsinghua University, Beijing, 100084 China; 2 Beijing Advanced Innovation Center for Structural Biology, Tsinghua University, Beijing, 100084 China; 3 State Key Laboratory of Membrane Biology, School of Life Sciences, Tsinghua University, Beijing, 100084 China

**Dear Editor,**

Voltage-gated sodium (Na_V_) channels are membrane proteins that are responsible for the propagation of action potentials in mammals by mediating Na^+^ influx in excitable cells such as nerve and muscle. In human, Na_V_ channels are therapeutic targets as their mutations contribute to many diseases. Structures of prokaryotic Na_V_ channels, e.g., Na_V_Ab (Payandeh et al., 2011), Na_V_Rh (Zhang et al., 2012) and Na_V_Ms (Mccusker et al., 2012), were successively determined in the past years. Recently, the cryo-EM structures of two eukaryotic Na_V_ channels were reported (Shen et al., 2017; Yan et al., 2017). Na_V_ channels contain 24 transmembrane helices, where each six consecutive segments (S1–S6) compose a homologous repeat. Segments S1–S4 form four voltage-gated domains (VSDs) respectively, while segments S5–S6 jointly constitute the pore domain (PD) that allows ion conduction. The intervening sequences between S5 and S6 segments constitute the selectivity filter (SF), which determines the ion selectivity. Unlike the homotetrameric architecture of prokaryotic Na_V_ channels, eukaryotic Na_V_ channels have the four homologous repeats connected within a single polypeptide, thus leading to asymmetric amino acid distribution in the SF (Shen et al., 2017; Yan et al., 2017). Particularly, the constriction site of the SF in eukaryotic Na_V_ channels consists of four different residues, Asp, Glu, Lys and Ala (or DEKA), each contributed from one homologous repeat. Notably, the DEKA residues are highly conserved in mammalian Na_V_ channels and are essential for the selection of permeating cations (Schlief et al., 1996; Sun et al., 1997), e.g., Na^+^ vs. K^+^ ions. Specifically, K is indispensable whereas at least one of D and E should be present to sustain the proper Na^+^/K^+^ selectivity. In contrast, the corresponding site contains four identical residues (EEEE or SSSS) in prokaryotic Na_V_ channels (Payandeh et al., 2011; Mccusker et al., 2012; Zhang et al., 2012).

Molecular dynamics (MD) simulations have been extensively employed to study the ion permeation, ion selection, voltage gating and ligand binding of Na_V_ channels (Li and Gong, 2015). However, nearly all previous simulations of Na_V_ channels started from prokaryotic structures, therefore incapable of elucidating the unique eukaryotic properties arising from the asymmetric amino acid distribution, for instance, the role of DEKA in cation selection. In the few studies employing eukaryotic structural models that were constructed from prokaryotic Na_V_ structures (Xia et al., 2013; Mahdavi and Kuyucak, 2015; Ahmed et al., 2017), authority of main conclusions was weakened by the non-negligible structural differences between the SFs of prokaryotic and eukaryotic Na_V_ channels. Recently, the structure of Na_V_PaS from American cockroach was determined by cryo-EM (Shen et al., 2017). Particularly, the SF region of this structure model is clearly defined from the electron density map (Fig. S1A), which allows the mechanistic study of ion permeation and ion selection in eukaryotic Na_V_ channels with atomistic details.

In this work, we adopted the PD of Na_V_PaS to study the ion-conducting pattern and Na^+^/K^+^ selectivity in the SF of eukaryotic Na_V_ channel by MD simulations. We observed distinct ion permeation behaviors between Na^+^ and K^+^ ions through equilibrium simulations. Application of a self-developed novel 3D ridge detection algorithm on the equilibrium trajectories, for the first time, allowed us to find an asymmetric path for Na^+^ ions to permeate through the SF region of a eukaryotic Na_V_ channel. Free-energy calculations along the path and at specific favorable ion binding sites explains the origin of Na^+^/K^+^ selection in Na_V_PaS.

To reduce computational complexity, we removed all extracellular domains from the PD (Fig. 1A) and built the system as shown in Fig. S1B. Because the vacant central cavity was rapidly filled by water molecules in the pre-equilibrations (Fig. S2A), the protein structure was stable in the ~300 ns equilibrium simulations in the presence of 150 mmol/L NaCl and KCl, respectively. Notably, the root-mean-square-fluctuation (RMSF) exceeds 2 Å only at the fragments that fall outside the lipid bilayer (Fig. S2B and S2C). After disregarding the most flexible intracellular fragment (residue 429–435) of TM6 (Fig. S2D), the root-mean-square-deviation (RMSD) converges to a plateau of <2.5 Å in both systems (Fig. S3). Therefore, removal of VSDs and extracellular domains from the PD introduced no negative impacts on the structural stability of the simulated protein, particularly the SF region that we focused on for the study of ion conduction and selection. Despite the general stability of the SF regions, the DEKA residues exhibit rapid side-chain movements (Fig. S4A and S4B). Interestingly, side chain of the Lys (K) residue protrudes towards the central cavity for most of the simulation time, which is consistent with the simulation on the homology model of human Na_V_1.5 (Ahmed et al., 2017) albeit different from the observation on the DEKA derivative of Na_V_Rh (Xia et al., 2013). We estimated the upper and lower boundaries of the DEKA region in the Z-axis (Z = 0 stands for position of the center of mass of the whole protein) from the mean and standard deviation of Z-coordinates of specific side-chain heavy atoms (C_γ_, C_δ_, N_ζ_ and C_β_ atoms of D, E, K and A residues, respectively) in the trajectories (Fig. S4A and S4B).

**Figure 1:**
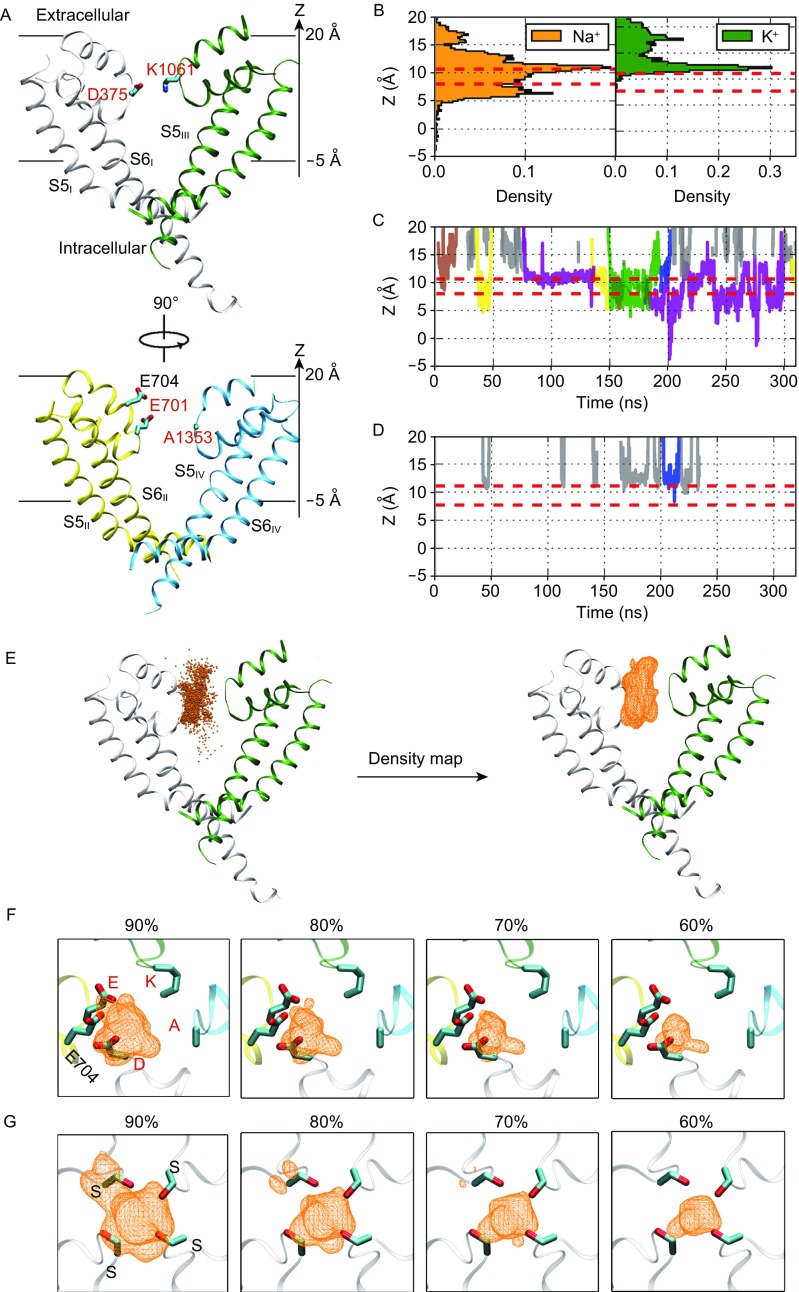
**Na** ^**+**^ **ions prefer to permeate in an asymmetric manner whereas K** ^**+**^ **ions are reluctant to enter the SF of Na**_**V**_ **Pas**. (A) Structure of the PD of Na_V_PaS. The side chains of residue Glu704 as well as the DEKA residues (Asp375, Glu701, Lys1061 and Ala1353) are shown as sticks. Four homologous repeats (I–IV) are colored in grey, yellow, green and cyan, respectively. (B) Probability distributions of Na^+^ and K^+^ ions residing in the SF. Boundaries of the DEKA region are labeled by red dotted lines. (C and D) Time-dependent ion trails of Na^+^ (C) and K^+^ (D) ions. Trails of ions that have ever entered the DEKA region are displayed in different colors to distinguish the ion identity, while the others are shown in grey. (E) 3D probability density map as well as the contour envelops (in orange wireframes) are obtained from the positions of Na^+^ ions that entered the SF of Na_V_ channels. (F) Top views of the contour envelopes of probability density map covering 90%, 80%, 70% and 60% of density points in Na_V_PaS. (G) Top views of the contour envelopes of probability density map covering 90%, 80%, 70% and 60% of density points in Na_V_Rh

We then analyzed the permeation behaviors of Na^+^ and K^+^ ions through the SF. For simplicity, we only counted the ions that stayed within the SF (−5 Å < Z < 20 Å) for at least 1 ns. Probability distributions clearly suggest that Na^+^ ions are broadly distributed throughout the SF vestibule, while K^+^ ions mainly linger outside of the DEKA region (Fig. 1B). Consistently, time-dependent ion trails show that Na^+^ ions frequently cross the DEKA region and sometimes permeate into the central cavity (Fig. 1C). In contrast, K^+^ ions seldom pass the extracellular boundary of the DEKA region and leave quickly even after occasional entrance (Fig. 1D). We then calculated the residence time of ions staying below the extracellular boundary of the DEKA region. As expected, Na^+^ ions exhibit a broad distribution with a long tail, approaching the maximum of 24 ns (Fig. S4C), whereas K^+^ ions can stay for at most 400 ps (Fig. S4D). Conclusively, the above observations suggest that our equilibrium simulations successfully reproduce the strict selection of Na^+^ against K^+^ ions by the SF of Na_V_PaS.

Considering the sufficient sampling of Na^+^ ions within the SF in the equilibrium simulation, we recorded positions of all valid Na^+^ ions (staying in the SF for at least 1 ns) and then generated the 3D probability density map for these positions (Fig. 1E). To facilitate visualization, contour envelops covering 90%, 80%, 70% and 60% of the density points were presented along with the cryo-EM structure of Na_V_PaS. As shown in Figure 1F, with the decrease of included density points, the contour envelop shifts gradually towards D and E in the DEKA loci, which strongly indicates that Na^+^ ions prefer to enter the SF of Na_V_PaS in an asymmetric manner (along the side wall near D and E). As a control, we simulated the Na^+^ permeation in a prokaryotic Na_V_ channel, Na_V_Rh, and produced the ion density map following the same protocol. Notably, Na_V_Rh presents a homotetrameric architecture with four Ser residues (SSSS) at the constriction site of the SF and four Glu residues (EEEE) at the entrance to the SF. Despite the reported tiny structural asymmetry, contour envelopes of the probability density map are always distributed around the central axis of the pore (Fig. 1G). Therefore, Na^+^ ions are likely to have remarkably different permeation behaviors in prokaryotic and eukaryotic Na_V_ channels, and simulations on eukaryotic channels such as Na_V_PaS may provide more valuable insights to the mechanism of Na_V_ channels in higher organisms.

Given the asymmetric probability density map of Na^+^ ions in the SF of Na_V_PaS, we then developed a novel 3D ridge detection algorithm to locate the most probable permeation path of Na^+^ ions from the extracellular solution to the central cavity (Fig. 2A). As a well-developed technique in computer image processing, ridge detection was introduced to capture the axis of elongated objects in 2D images and has been widely used in detecting roads in aerial images as well as analyzing medical images. Recently, this method was extended to 3D space to identify the scattered points along the neuron axons and dendrites in the transmission electron micrographs (Mishchenko, 2009). Here, for the first time, we applied this technique to find the axes of elongated distributions in a 3D probability density map of ion positions obtained from an equilibrium simulation, which intrinsically correspond to the highly probable trails of ion motion. Specifically, we chose the *γ*-normalized derivatives method of 2D ridge detection introduced by Lindeberg (Lindeberg, 1998) and developed the mathematical derivation for its 3D application (see Supplementary Materials for details). Moreover, unlike previous 3D studies that could only find scattered points of short ridges, we used isometric mapping to locate long and continuous curves among all identified ridge points (see Supplementary Materials for details), which could better describe the ion permeation path that span from the extracellular space to the central cavity. Theoretically, our 3D ridge detection method could be applied to process any 3D signals. As a validation, in an application to a medium-resolution cryo-EM electron density map, the long and continuous curves identified by our method agree well with the directions of protein backbone traces in helical regions that exhibit relatively strong electron-density signals (Fig. S5).

**Figure 2:**
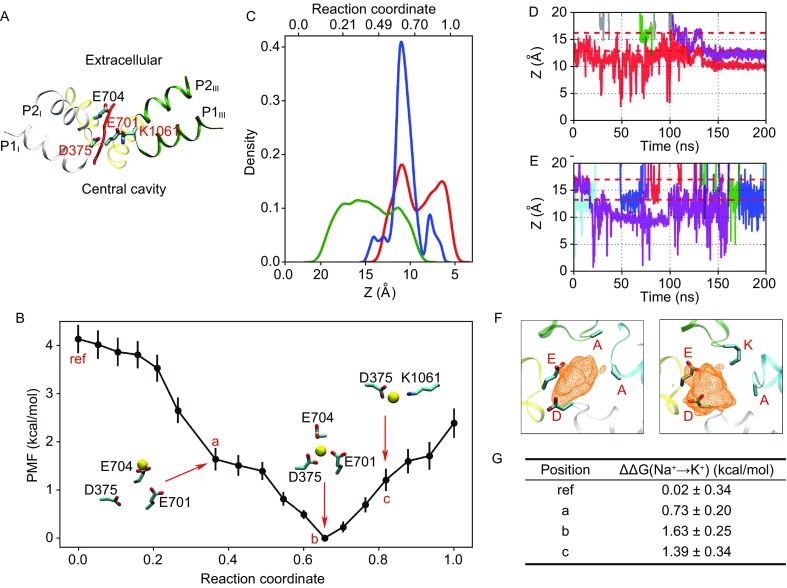
**Calculations along the most probable ion permeation path imply the origin of Na** ^**+**^ **/K** ^**+**^ **selectivity in Na**_**V**_ **Pas**. (A) The permeation path (red curve) of Na^+^ ions from the extracellular bulk to the central cavity identified by the 3D ridge detection algorithm. Only P-loops of homologous repeats I-III are shown for clarity. (B) The PMF profile along the identified permeation path. Three interesting windows in the umbrella sampling calculations are labeled as a, b and c on the curve, with representative structures shown aside. Representative structures of these three windows as well as a reference window (labeled as ref) were chosen for free energy perturbation calculations. (C) The probability density function of Na^+^ coordination by Glu704 (green), Asp375 (red) and Glu701 (blue) in all windows of the umbrella sampling calculations, respectively. Relationship between window Z-coordinates and reaction coordinates is shown in Fig. S7. (D and E) Time-dependent ion trails of Na^+^ (D) and K^+^ (E) ions in the DEAA derivate. Boundaries of the DEAA region (red dotted lines) were estimated in a similar approach to Fig. S4A and S4B. Trails of ions that have ever entered the DEAA region are displayed in different colors to distinguish the ion identity, while the others are shown in grey. (F) Top views of the probability density maps of Na^+^ ions in the DEAA (left) and DEKA (right) proteins. (G) FEP calculations for evaluating Na^+^/K^+^ selectivity at four positions defined in (B)

In this work, when analyzing the probability density map of Na^+^ ions, we identified only one long and continuous curve that passes through the SF (Fig. 2A), which should correspond to the most probable ion permeation path. Besides strength, our ridge detection algorithm also reported a width for each ridge point (see Supplementary Materials for details), which depicts the ion positional fluctuation in the direction perpendicular to the path. As shown in Figure S6, contour envelopes covering 90%, 80%, 70% and 60% of the density points are all distributed around the ridge, which supports the “most probable” property of the identified path. As shown in Movie S1, when permeating along the identified path, Na^+^ ions have to pass through D (Asp375) and E (Glu701) in the DEKA region. Notably, a highly conserved acidic residue, Glu704 in repeat II, is also located around the path and is actively involved in ion coordination. We want to emphasize that the most probable ion permeation path identified here is unlikely to locate using conventional simulation methods. In the simulation of prokaryotic Na_V_ channels, the structural symmetry of these channels allows people to simply use the vertical axis to roughly describe the ion permeation events. Such simple approaches become powerless in the analysis of Na_V_PaS, where the ions clearly enter the SF in an asymmetric manner. Moreover, the string method with swarms of trajectories (Pan et al., 2008), which is frequently used in locating the minimum free energy reaction paths through swarms of iterative simulations, requires that the termini of the path should be energetically stable. However, the termini of the ion permeation path belong to the aqueous extracellular space and the central cavity, both of which lack stable ion binding positions. Therefore, the combination of 3D ridge detection and equilibrium simulations as developed in this work successfully overcomes the deficiency of previous methods and would thus become an effective approach in the analysis of ion permeation behaviors in eukaryotic Na_V_ channels.

Subsequently, we calculated the free energy profile along the identified ion permeation path for a single Na^+^ ion using umbrella sampling (US) (see Supplementary Materials for details). Specifically, the path was evenly divided into windows along the Z-axis, with the window center ranging from 17.5 Å to 5.5 Å. The path was further extended at the extracellular side by appending a number of additional windows (Z from 22.5 Å to 18.5 Å) to consider the initiation of ion permeation from the bulk extracellular solution. Within the final path, reaction coordinates are roughly linearly correlated with Z-coordinates of window centers (Fig. S7). In each window, the starting conformation was chosen from the equilibrium simulation, and a cylindrical restraint was applied to ensure sampling around the path based on the average ridge width within the window (Table S1). Sufficient sampling overlaps between all nearby windows and convergence of the free energy curves obtained at successive 5 ns intervals both support the rigor of our calculation (Fig. S8A and S8B). Figure 2B shows the final potential of mean force (PMF), where a favorable free energy well of ~4 kcal/mol (position b in Fig. 2B) explains the attraction of extracellular Na^+^ ions into the SF region as observed in the equilibrium simulation. Previous simulation study on the homology model of Na_V_1.4 (Mahdavi and Kuyucak, 2015) reported an ion binding site at the similar position with comparable well depth, but suggested the presence of a extracellular free energy barrier of ~3 kcal/mol. In contrast, our PMF curve is barrier-less at the extracellular side, and this difference reinforces the importance of simulation study and free energy calculation using an authentic eukaryotic structure.

Structural analysis of snapshots in all US windows implies that three acidic residues, Glu704, Asp375 (D) and Glu701 (E), are actively involved in coordination with the Na^+^ ion permeating along the path (as defined by carboxylate-ion distance <3.1 Å (Carnevale et al., 2011)). As shown in Figure 2C, the ion first interacts with Glu704 in repeat II (see the representative structure at position a in Fig. 2B), which then acts as a relay to present the ion to Asp375 (repeat I) and Glu701 (repeat II) through side-chain swinging. At the constriction site, the Na^+^ ion is favorably coordinated by the three carboxylate groups of Glu704, Asp375 and Glu701 simultaneously (see the representative structure at position b in Fig. 2B), which explains the favorable free energy well in the PMF curve. When passing through the inwardly protruding Lys1061 (K of the DEKA loci) side chain, the ion is still partially coordinated by Asp375 (see the representative structure at position c in Fig. 2B), which compensates the electrostatic repulsion between the Na^+^ ion and the Lys side chain. Hydration analysis along the ion permeation path suggests that the Na^+^ ion is never fully dehydrated even at the lowest free-energy position. Consistent with previous studies (Carnevale et al., 2011), loss of hydration could be effectively compensated by the carboxylate groups, and the overall coordination number retains at 5–6 (Fig. S9).

Starting from the three representative US windows shown in Fig. 2B (positions a, b and c), we conducted the free energy perturbation (FEP) calculations (see Supplementary Materials for details) to evaluate the Na^+^/K^+^ selectivity (Fig. S10). Specifically, the Na^+^/K^+^ selectivity was quantified as the relative binding affinity ΔΔG(Na^+^→K^+^), which indicates Na^+^ preference when positive and vice versa. The extracellular US window of position ref in Fig. 2B was taken as the control. As shown in Figure 2G, the position ref exhibits no Na^+^/K^+^ selectivity, while the other three positions all show marked Na^+^ preference (ΔΔG(Na^+^→K^+^) >0.7 kcal/mol). Particularly, at the lowest free-energy position (position b), coordination of the cation by three carboxylate groups imposes the strongest Na^+^/K^+^ selectivity. Given the FEP results, hypothetical PMF values of K^+^ ion permeating along the same path could be estimated at the four positions (Fig. S11), which explains the short residence time of K^+^ ions as observed in the equilibrium simulation. Considering that the ion is coordinated by carboxylate groups at positions of marked Na^+^/K^+^ selectivity (positions a, b and c), Na^+^/K^+^ selectivity in eukaryotic Na_V_ channels is likely to arise from the coordination by conserved acidic residues, consistent with our previous proposition (Xia et al., 2013).

Lys1061 (K of the DEKA loci) also plays an essential role by restricting the ion to permeate along the side wall of multiple acidic residues. We speculate that mutation of this residue will abolish or weaken the electrostatic repulsion, which will allow the ion to permeate along the side wall opposite to D and E given the spacious SF vestibule of eukaryotic Na_V_ channels and thus will impair the Na^+^/K^+^ selectivity. To validate this hypothesis, we generated the DEAA mutant of Na_V_PaS (by mutating Lys1061 to Ala) and then simulated the ion permeation behaviors for this derivative. In contrast to the wide-type DEKA protein, both Na^+^ and K^+^ ions can enter the SF of the DEAA derivative without difficulty, which indicates the disruption of Na^+^/K^+^ selectivity by the Lys-to-Ala single mutation (Fig. 2D and 2E). Furthermore, comparison on probability density maps of Na^+^ ions that have entered the SF region also suggests that ion permeation becomes less asymmetric in the absence of Lys side chain (Fig. 2F). These additional simulations thus support our speculation about the essential role of Lys in the Na^+^/K^+^ selectivity of Na_V_PaS, and are consistent with results of previous electrophysiological experiments on eukaryotic Na_V_ channels (Schlief et al., 1996; Sun et al., 1997).

In conclusion, our simulations on the PD of Na_V_PaS show expected difference between the permeation behaviors of Na^+^ and K^+^ ions. Na^+^ ions can favorably bind and permeate through the SF in an asymmetric manner, which allows the identification of an ion permeation path. Free-energy calculation along the path and at specific ion binding sites explain the origin of Na^+^/K^+^ selection in Na_V_PaS. Our results thus shed light on the mechanism of ion permeation and ion selection in eukaryotic Na_V_ channels. Furthermore, we developed a novel method to locate the ion permeation pathway from MD trajectories. The combination of 3D ridge detection and MD simulations is likely to become an informative and effective method in the analysis of ion permeation in eukaryotic Na_V_ channels. Nevertheless, all observations and conclusions were obtained purely from molecular simulations, and therefore still await experimental validation

## Electronic supplementary material

Below is the link to the electronic supplementary material.

Supplementary material 1 (PDF 3371 kb)
