# Supplementary material for: Simulating the ion permeation and ion selection for a eukaryotic voltage-gated sodium channel NaVPaS
Source: Protein Cell. 2018 Mar 12;9(6):580–5. doi: 10.1007/s13238-018-0522-y (PMC5966359; doi:10.1007/s13238-018-0522-y)
Supplement: Supplementary file 1 — Supplementary material 1 (PDF 3371 kb) [file 13238_2018_522_MOESM1_ESM.pdf]

Materials and Methods

MD simulations

The initial structure in MD simulations was taken from NavPaS, a cryo-EM structure solved at a resolution of 3.8 Å (PDB ID: 5X0M). After removing all VSDs and extracellular domains of the PD, the remaining structure of NavPaS was embedded in a hydrated palmitoyl-oleoyl-phosphatidyl-choline (POPC) bilayer. After solvation and ionization, the number of atoms reached ~84000. The CHARMM36 force field with CMAP correction (MacKerell Jr et al., 1998; Best et al., 2012) and with NBFIX (Luo and Roux, 2010) applied on interactions between Na^+/K^+ and Cl^- ions was used in all simulations. Simulations were performed by NAMD 2.10 and NAMD 2.12 (Phillips et al., 2005).

Equilibrium simulations

The extracellular domains of PD were removed to reduce the computational complexity, and the retained residues included 244-281, 361-435, 625-663, 687-746, 968-1002, 1047-1072, 1087-1121, 1283-1321, 1339 -1363 and 1385-1421. TIP3P model was adpoted to describe water. The van der Waals interaction was smoothly switched to zero from 10 to 12 Å and was cutoff at distance beyond 12 Å. Electrostatic interaction was calculated by the particle mesh Ewald (PME) method (Essmann, 1995), with period boundary conditions applied in all directions. The scaled1-4 exclusion policy was set to specify the atomic interactions within 3 chemical bonds. All simulations were controlled at 310 K by the Langevin thermostst (Tu et al., 1995) and at 1 atm by the Langevin piston (Martyna et al., 1994; Feller et al., 1995), respectively. During the simulations, the C_α atoms of residues 244, 625, 968 and 1283 (the N-terminal one for each TM5

helix) were restrained harmonically (with a force constant of 0.01 kcal/mol/Å^2) to eliminate the negative influence of removing VSDs.

Both systems (in NaCl and KCl) were pre-equilibrated in the following steps: (1) all atoms were fixed except the lipid tails for 2000 steps of energy minimization and 0.5 ns of equilibration; (2) the protein were restrained by a harmornic restraint of 1kcal/mol/Å^2 for 2000 steps of energy minimization and 1 ns of equilibration; (3) the restraint was applied to all protein non-hydrogen atoms for 1 ns of equilibration; (4) the restraint was applied to the protein backbone for 1ns of equibration; (5) a restraint of 0.1 kcal/mol/Å^2 was applied to the C_α atoms of the protein for 1 ns of equilibration; (6) a restraint of 0.01 kcal/mol/Å^2 was applied to the C_α atoms of the protein for 1 ns of equilibration; (7) a restraint of 0.01kcal/mol/Å^2 was applied to the C_α atoms of residues 244, 625, 968 and 1283 for 10 ns of equilibration. The time step was set to 2 fs and all hydrogen atoms were restrained by the SETTLE algorithm (Miyamoto and Kollman, 1992). Steps (2)-(7) were performed in NPT ensembles, while Step (1) in NVT ensembles. In Step (2)-(6), the ions were restrained at least 10 Å away from the SF using a flat-bottom potential. After the pre-equilibrations, production simulations were performed for both systems for more than 300 ns in NPT ensembles, with the area of membrane held as a constant.

PMF calculations

The PMF profile was calculated using the umbrella sampling (US) (Torrie and Valleau, 1977; Chen and Chung, 2012) method along the identified ion permeation path. The identidied path was evenly divided into windows along the Z-axis, with additional windows appended at

the extracellular region. A total of 18 windows were set up with Z-coordinate spanning from 22.5 Å to 5.5 Å. The initial structures of all US windows were taken from the equilibrium simulation. In the XY-plane, a force constant of 10 kcal/mol/Å^2 was applied as long as the ion escaped the cylinder around the path defined by the average ridge width with the window. The detailed cylindrical restraints are listed in Table S1. The other parameters were the same as the equilibrium simulation. Each window was simulated for at least 30 ns, until the sufficient overlaps of nearby windows and convergence of free energy curves were guaranteed (see Figure S8). To avoid artifacts, snapshots of the first 5 ns were not included in the estimation of PMF curves using the weighted histogram analysis method (WHAM) (Habeck, 2012; Hub et al., 2015).

FEP calculations

To compare the binding affinities of Na^+ and K^+ ions, we used the FEP method (Zwanzig, 1954; Kollman, 1993) to evaluate the relative binding affinity at four sites. Acoording to the protocol of FEP method in Figure S10, the ΔΔG ($Na^+ \rightarrow K^+$) can be derived using the following equation,

$$\begin{aligned} \Delta\Delta G(Na^+ \rightarrow K^+) &= \Delta G_{binding}(Na^+) - \Delta G_{binding}(K^+) \\ &= \Delta G_{site}(Na^+ \rightarrow K^+) - \Delta G_{bulk}(Na^+ \rightarrow K^+) \end{aligned}, \quad (1)$$

where the $\Delta G_{bulk}(Na^+ \rightarrow K^+)$ was estimated in a water box of 30×30×30 Å^3 for three times in both forward and backward directions. Then the calculations of $\Delta G_{site}(Na^+ \rightarrow K^+)$ at four sites were performed also bi-directionally. The starting structures were taken from trajectories of the US simulations. At the end of simulations, forward and backward calculations showed sufficient

overlaps between the probability distributions of energy perturbation in all windows, and the difference between forward and backward free energy values were < 1.5 kcal/mol, both of which indicated convergence of calculation. The Bennett acceptance ratio (BAR) estimator (Bennett, 1976; Pohorille et al., 2010) was adopted to estimate the mean and standard deviation of free energy changes by combing the forward and backward result. The details of FEP results are shown in Table S2. In all FEP calculations, the soft core potential (Zacharias et al., 1994) was adopted to avoid "end-point catastrophes" and intra-molecular interactions were decoupled from the FEP calculations. The time step was set to 1 fs and 2 ns of equilibrium was performed before FEP calculations. 5000 steps of equilibration was conducted for each window. Details of the stratification are listed in Table S3. A force constant of 10 kcal/mol/Å^2 was applied when the ion sampled outside the 1 Å sphere centered at the initial position. The positions of Na^+ and K^+ ions was constrained to overlap by a strong force constant of 10 kcal/mol/Å^2. The other parameters are identical to the equilibrium simulation.

All MD simulations are briefly summarized in Table S4.

3D ridge detection algorithm

Ridge detection is a well-developed tool to identify elongated object in images. It has been widely used in 2-dimensional analysis of aerial (Laptev et al., 2000) and medical (Sato et al., 1998) images. There are also studies to extend the usage to 3-dimensional data like magnetic resonance images (Frangi et al., 1999). In this study, we extended the γ-normalized scale-space

ridge detection to 3-dimensional probability density map based on the 2-dimensional method introduced by Lindeberg (Lindeberg, 1998).

Firstly, in order to employ the ridge detection, we reformatted our ion position data as regular grid data, like a 3-dimensional image. Here, we used the kernel density estimation (KDE) to build the probability density function (PDF) of the ion positions. KDE (Rosenblatt, 1956; Parzen, 1962) estimates the probability density based on a finite data sample. The relative positions from each sample position to the estimated point were calculated and the weights were then determined by a kernel function. When the estimated point is far away from the sample data, a lower weight is assigned in general. Thus, KDE could create a smooth density map that has higher density at locations with more sample points surrounded. Results of KDE are strongly influenced by the bandwidth (*h*) selection (Turlach, 1993; Bashtannyk and Hyndman, 2001). In this work, we used Gaussian kernel (*K*) and an automatic bandwidth determination method, the Silverman's Rule (Silverman, 1986), to select the bandwidth. The scientific Python package SciPy (Jones et al., 2014) was utilized to perform the kernel density estimation, following

$$\hat{f}(x) = \frac{1}{n}\sum_{i=1}^{n} K_h\left(x - x_i\right) = \frac{1}{nh}\sum_{i=1}^{n} K\left(\frac{x - x_i}{h}\right). \tag{2}$$

Secondly, we used the *γ*-normalized scale-space ridge detection to locate the ridge points based on the estimated density map. Ridge point is an extended conception of the local maximum. In an n-dimension space, local maximum points are required to be locally maximal in all directions, i.e. in all n dimensions, while the ridge points are only required to be locally maximal on an n-1 dimension sub-space. Mathematically, the ridge points in 3 dimension are

defined as below. For any point (x_0, y_0, z_0), we transfer the (x, y, z)-system to a local (p, q, r)-system aligned to the principal curvature directions of the density function. The position system is transformed by performing eigendecomposition of the local Hessian matrix (H):

$$\mathbf{H} = \begin{bmatrix} f_{xx} & f_{xy} & f_{xz} \\ f_{xy} & f_{yy} & f_{yz} \\ f_{xz} & f_{yz} & f_{zz} \end{bmatrix} = \begin{bmatrix} \mathbf{v_p} & \mathbf{v_q} & \mathbf{v_r} \end{bmatrix} \begin{bmatrix} f_{pp} & 0 & 0 \\ 0 & f_{qq} & 0 \\ 0 & 0 & f_{rr} \end{bmatrix} \begin{bmatrix} \mathbf{v_p^T} \\ \mathbf{v_q^T} \\ \mathbf{v_r^T} \end{bmatrix}, \quad (3)$$

where f_{**} is the corresponding second order derivative and the v_* is the curvature direction. A local maximum point should be maximal on all p, q and r direction:

$$\begin{cases} f_p = 0 \\ f_q = 0 \\ f_r = 0 \\ f_{pp} < 0 \\ f_{qq} < 0 \\ f_{rr} < 0 \end{cases} . \quad (4)$$

In order to get the maximum points in an n-1 dimensional sub-space, we suppose the curvature on p, q and r directions have the order of $f_{pp} \le f_{qq} \le f_{rr}$. The ridge point could thus be defined as:

$$\begin{cases} f_p = 0 \\ f_q = 0 \\ f_{pp} < 0 \\ f_{qq} < 0 \end{cases} . \quad (5)$$

By the definition, the local maximum points also meet the requirement for the ridge point.

In Lindeberg's study (Lindeberg, 1998), the scale space is defined as an additional dimension to the 2-dimensional image. Similarly, we define the scale space data as the original density data (f) convolved with a Gaussian function with scale factor σ (g_σ):

$$f_\sigma = f * g_\sigma$$
$$f_\sigma(x_0, y_0, z_0, \sigma_0) = \iiint f(x_0 + u, y_0 + v, z_0 + s) g_{\sigma_0}(-u, -v, -s)\ du\ dv\ ds . \quad (6)$$

Lindeberg has shown that the ridge detection is very sensitive to the scale selection and no single scale is appropriate for the entire image (Lindeberg, 1998). In order to address the problem, Lindeberg introduced the γ-normalized ridge detection to automatically select the optimal scale (Lindeberg, 1998). For the 3-dimensional extension, we firstly introduced the ridge strength $\mathcal{GL}$ for 3-dimensional cases:

$$\mathcal{GL} = f_{pp}^2 + f_{qq}^2 . \tag{7}$$

In order to endow the ability to determine the optimal scale automatically, we also adopted the γ-normalized method. $\mathcal{G}_{\gamma\text{-norm}}\mathcal{L}$ is defined as

$$\mathcal{G}_{\gamma\text{-norm}}\mathcal{L} = \sigma^{\gamma}\left(f_{pp}^2 + f_{qq}^2\right) . \tag{8}$$

The value of γ is carefully selected to guarantee that a standard Gaussian ridge could reach the maximum at the ridge real scale. Thus, γ is different for different ridge strength definition, and it is 4 for $\mathcal{G}_{\gamma\text{-norm}}\mathcal{L}$:

$$\left.\frac{\partial f_\sigma}{\partial \sigma}\right|_{\sigma=\sigma_{real}} = 0, \text{ when } \gamma = 4 . \tag{9}$$

Utilizing the γ-normalized ridge detection, we could locate the ridge points in the scale space with automatic scale selection.

Finally, we utilized the isometric mapping (Isomap) (Tenenbaum et al., 2000), a nonlinear dimensionality reduction method, to determine the final path. Isomap is a dimension reduction method trying to keep identical distance from each sample to the surrounding samples. The method first constructs a graph with each sample as a node. For each sample, the edge is connected to the surrounding sample node with length equal to the Euclidean distance, and the

distances between any two samples are defined as the shortest path length in the graph. Then, we perform the multidimensional scaling (MDS) (Borg and Groenen, 2005) to map our samples to a lower dimensional space. In our work, the first dimension explains more than 99.99% of the distance matrix, which indicates a significant 1-dimensional line-like manifold. Based on the first dimension mapping from Isomap, we could determine the path of the ion motion. Isomap calculation was performed using the Python package scikit-learn (Pedregosa et al., 2011).

An example python script to identify the path from a 3D probability density map has been attached at the end of the Supplementary Materials.

Supplementary figures

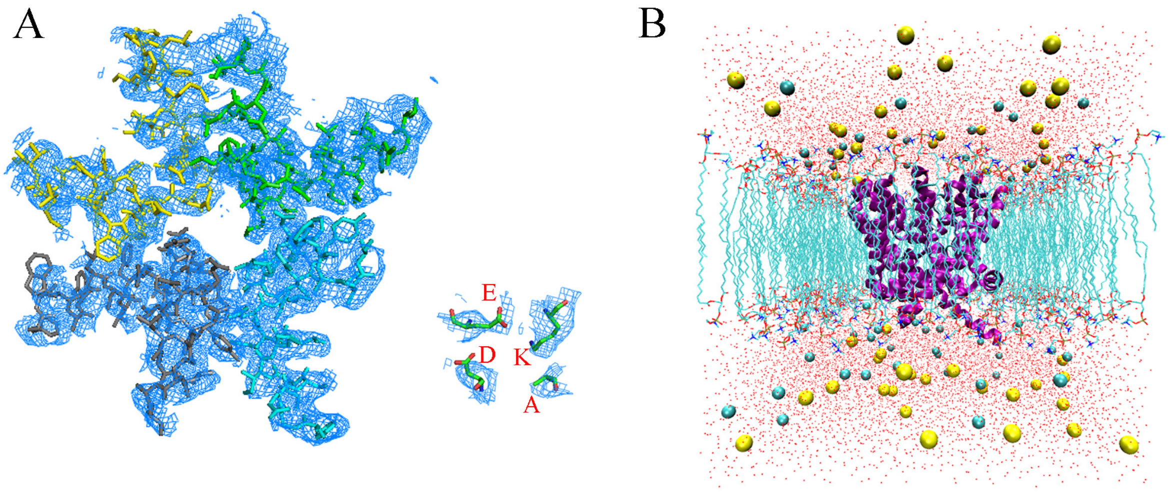

Figure S1. **(A)** The SF region of the NavPaS cryo-EM structure (top view) is well solved, where most side chains are determined from the electron density map with high confidence. The DEKA loci is zoomed in at the lower right corner. **(B)** Schematic representation of our simulation system (side view). The protein, lipids, waters and ions are shown in cartoon, line, CPK and VDW representations, respectively. Cations (Na^+ or K^+) and anions (Cl^-) are colored in yellow and cyan, respectively.

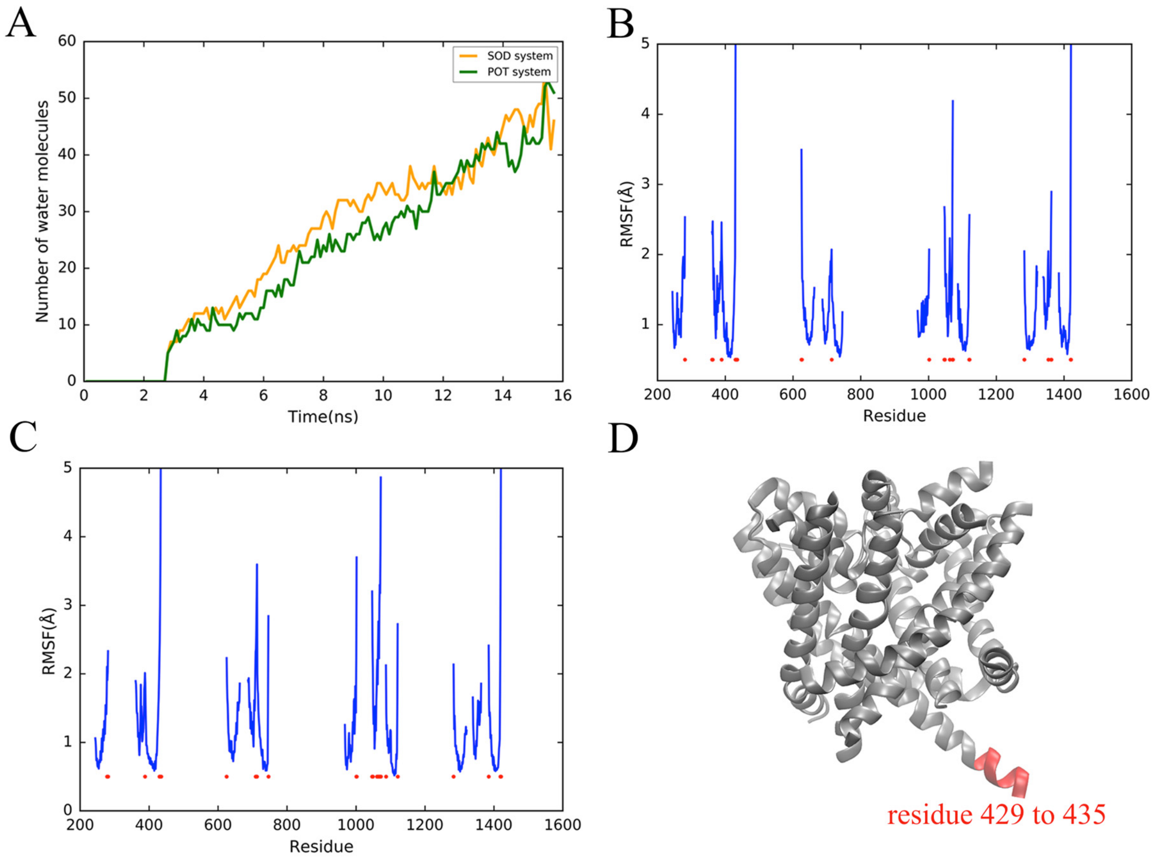

Figure S2. **(A)** Variation of the number of water molecules in the pre-equilibrations of simulations in NaCl (yellow) and KCl (green), respectively. **(B, C)** RMSF profiles for the equilibrium simulations in NaCl (**B**) and KCl (**C**), respectively. The regions with value > 2 Å are labeled by red dots at the bottom. **(D)** The flexible fragment (residue 429-435) of TM6 is highlighted in red within the cryo-EM structure of NavPaS.

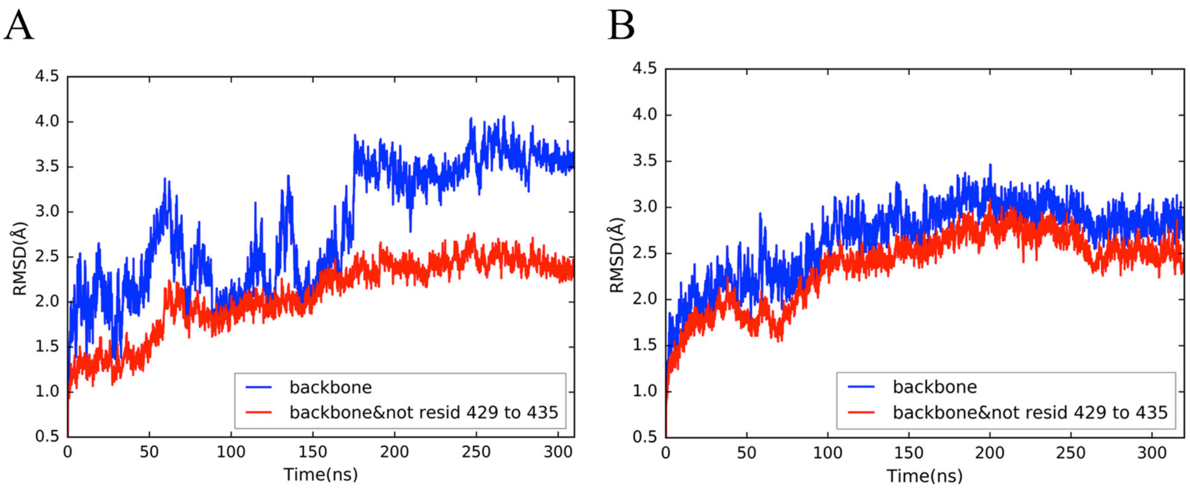

Figure S3. The time-dependent RMSD profiles for equilibrium simulations. **(A)** The backbone RMSD of simulation in NaCl. **(B)** The backbone RMSD of simulation in KCl. RMSD profiles were computed for the whole protein (blue) and the protein without residue 429-435 (red), respectively.

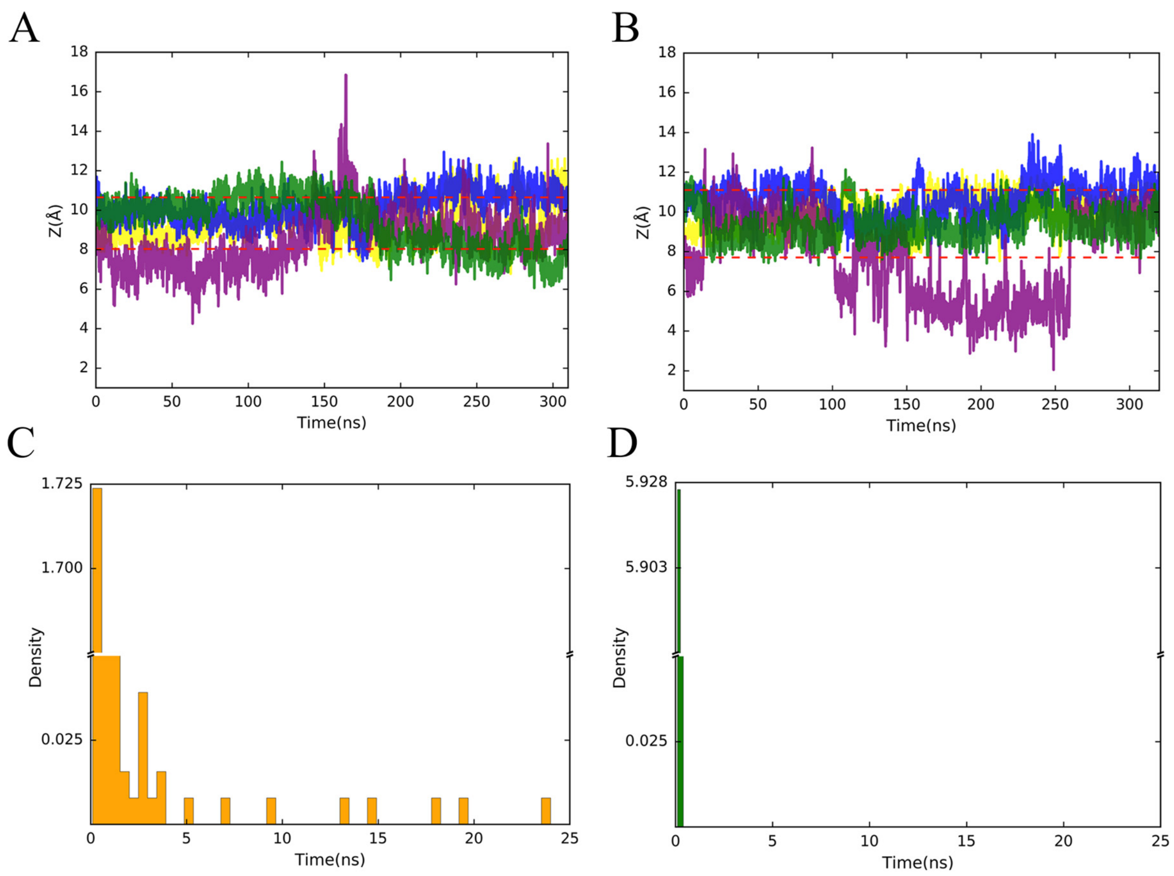

Figure S4. **(A, B)** The positional fluctuations of DEKA residues along the Z-axis in the equilibrium simulations in NaCl (**A**) and KCl (**B**), respectively. Evaluation was performed using the C_γ atom of Asp375 (yellow), C_δ atom of Glu701 (blue), N_ζ atom of Lys1061 (purple), and C_β atom of Ala1353 (green), respectively. Boundaries of the DEKA region were defined as mean ± standard deviation of the key atoms of D, E, K and A. **(C, D)** Probability distributions of the residence time for Na^+ (**C**) and K^+ (**D**) ions that ever passed the extracellular boundary of the DEKA region. The vertical axis is truncated here for clarity.

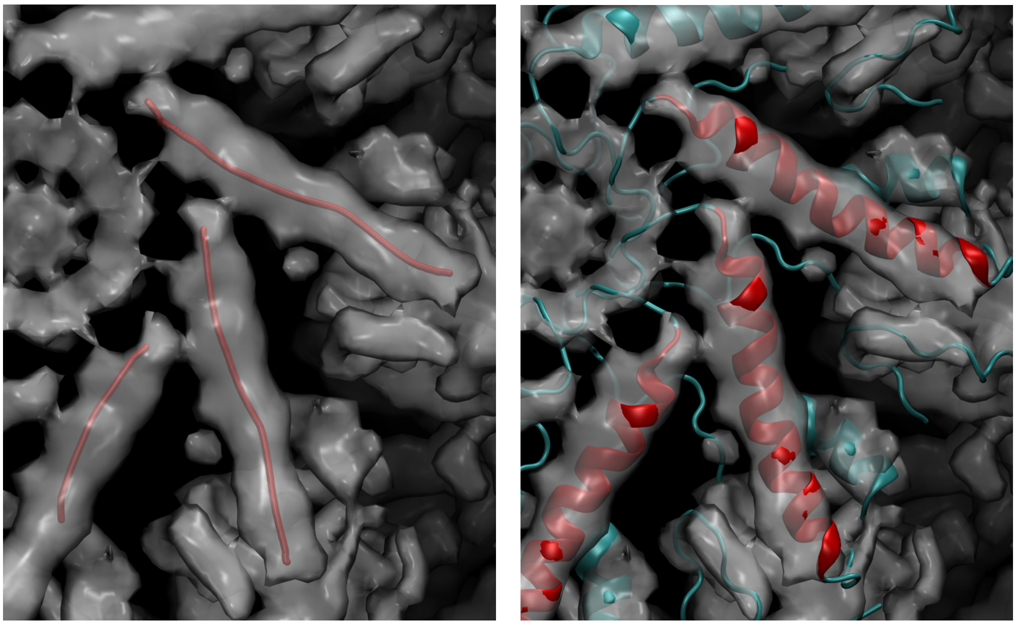

Figure S5. Application of our 3D ridge detection algorithm in identifying the axes of helices in the cryo-EM electron density map EMD-5137 (PDB id: 3LOS; resolution: 4.3 Å). The identified long and continuous curves (red in the left panel) agree well with the axes of helices (red in the right panel).

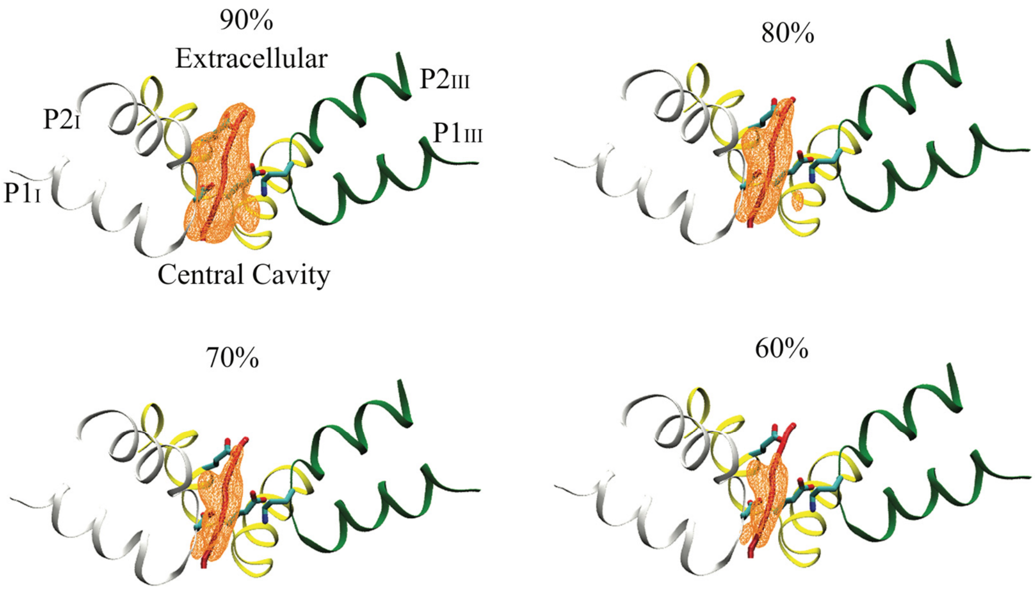

Figure S6. The identified path from the extracellular bulk to the central cavity with contour envelopes of probability density maps covering 90%, 80%, 70% and 60% of density points. The path is shown as a red thick line, and the density map is shown as orange wireframes. Only P-loops of homologous repeats I-III are shown for clarity.

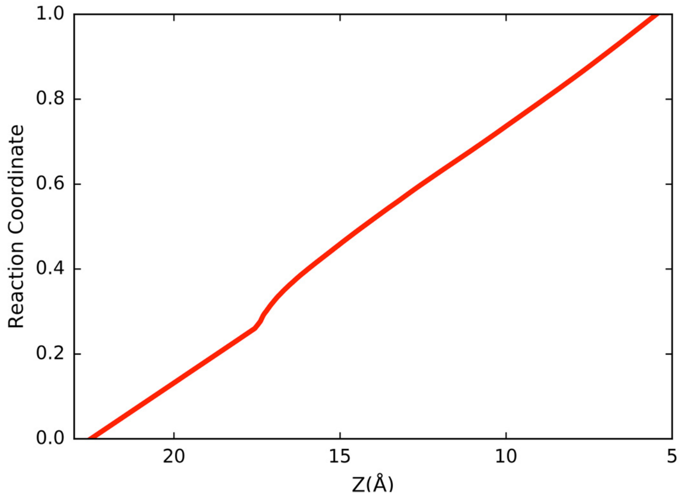

Figure S7. The reaction coordinate along the path is roughly linearly correlated to the Z-coordinate of US windows.

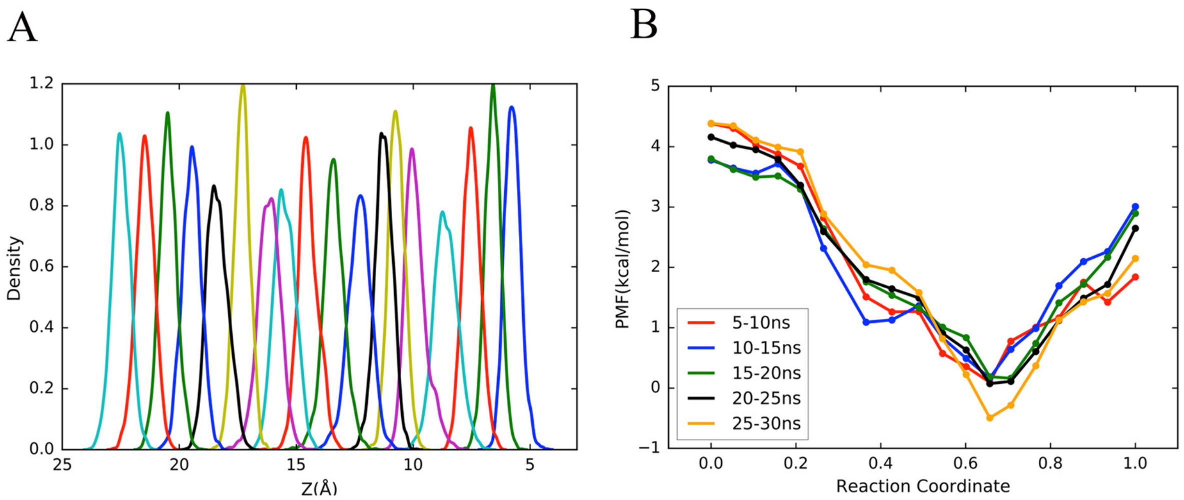

Figure S8. Validation of the rigor of US simulations. **(A)** Sufficient overlaps are present between the ion positional distributions of all nearby windows. **(B)** Free energy curves obtained from successive 5 ns intervals show good convergence.

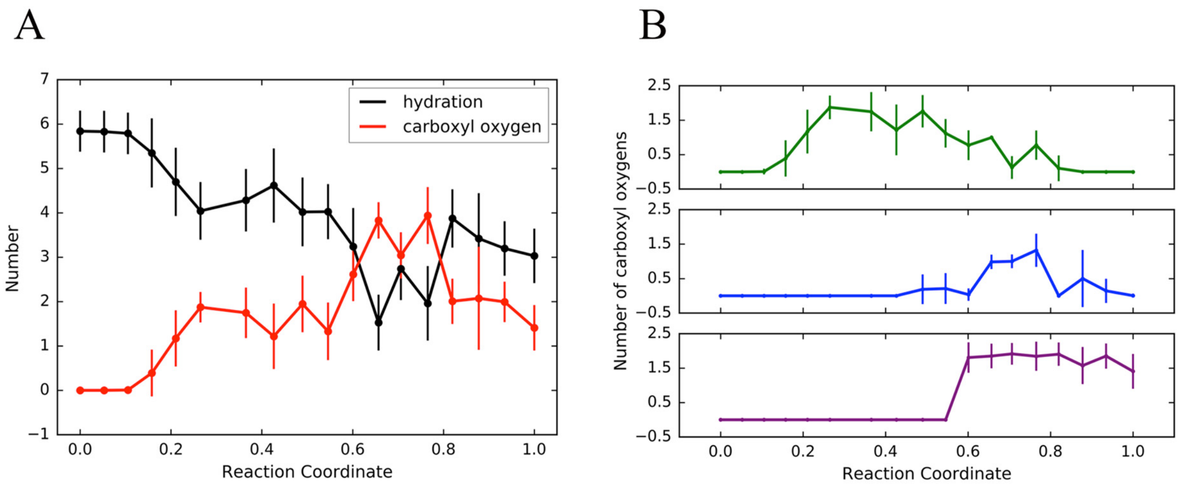

Figure S9. **(A)** The coordination number of the permeating Na^+ ion in each US window. Coordination was evaluated for waters (black) and carboxylate oxygen atoms (red), respectively. **(B)** Contribution of ion coordination by Glu704 (top), Glu701 (middle) and Asp375 (bottom) in each US window.

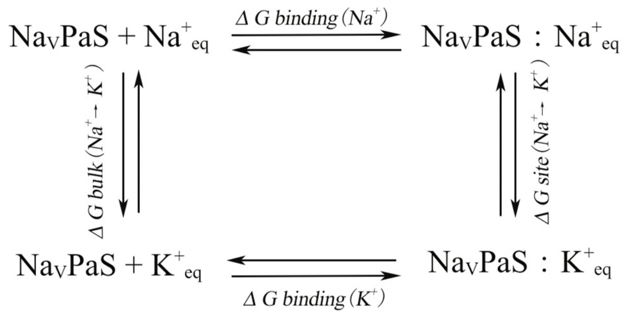

Figure S10. The protocol for FEP calculation. The $\Delta\Delta G(Na^+ \rightarrow K^+)$ equals the difference between $\Delta G_{site}(Na^+ \rightarrow K^+)$ and $\Delta G_{bulk}(Na^+ \rightarrow K^+)$, but could be obtained from FEP calculations (vertical reactions) according to the thermodynamic cycle diagram. $\Delta\Delta G(Na^+ \rightarrow K^+)$ is positive for Na^+ preference and is negative for K^+ preference.

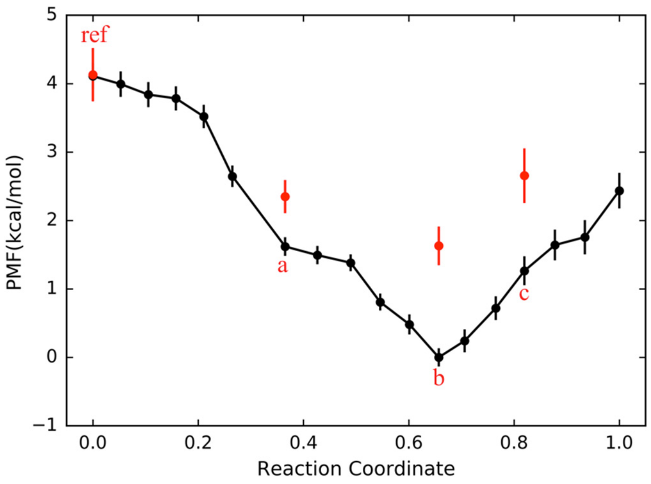

Figure S11. PMF values of the K^+ ion moving along the identified Na^+ permeation path could be estimated at four positions based on FEP results. The values of K^+ ions are colored in red.

Supplementary tables

Table S1. List of positional restrictions in the XY-plane in each US window.

window #	restraint XY (Å)	Z center (Å)	window #	restraint XY (Å)	Z center (Å)	window #	restraint XY (Å)	Z center (Å)
1	1.1	22.5	7	0.8	16.5	13	0.6	10.5
2	1.1	21.5	8	0.9	15.5	14	0.8	9.5
3	1.1	20.5	9	1.4	14.5	15	0.9	8.5
4	1.1	19.5	10	1.4	13.5	16	1.2	7.5
5	1.1	18.5	11	1.0	12.5	17	1.4	6.5
6	1.0	17.5	12	0.7	11.5	18	1.4	5.5

Table S2. Detailed results of FEP calculations.

position	$\Delta G_{bulk}(Na^+ \rightarrow K^+)$ (kcal/mol)			
	forward	backward	BAR estimator	average
In bulk	18.54	-18.52	18.62±0.11	18.64±0.11
	18.42	-18.46	18.52±0.13	
	18.51	-18.79	18.78±0.10	
	$\Delta G_{site}(Na^+ \rightarrow K^+)$ (kcal/mol)			
position	forward	backward	BAR estimator	average
ref	18.49	-18.47	18.70±0.07	18.66±0.32
	18.44	-18.38	18.62±0.07	
	18.50	-18.45	18.66±0.06	
a	19.11	-18.86	19.22±0.07	19.37±0.17
	19.31	-19.53	19.61±0.08	
	19.08	-19.07	19.29±0.07	
b	20.38	-19.19	19.99±0.03	20.27±0.23
	19.90	-20.18	20.28±0.04	
	20.06	-20.47	20.55±0.04	
c	19.97	-20.30	20.34±0.07	20.03±0.32
	19.66	-19.10	19.60±0.08	
	19.70	-20.03	20.16±0.07	

Forward: FEP calculation for the alchemical transformation from Na^+ to K^+. Backward: FEP calculation for the alchemical transformation from K^+ to Na^+. BAR estimator: comprehensive free energy estimated by the BAR estimator. $\Delta G_{bulk}(Na^+ \rightarrow K^+)$ was estimated in a water box of 30×30×30 Å^3.

Table S3. Stratification details in the FEP calculations.

λ	Δλ	Steps ($\times 10^4$)	λ	Δλ	Steps ($\times 10^4$)
[0,0.0001]	0.0001	4	[0.49,0.70]	0.0025	4
[0.0001,0.0005	0.0004	4	[0.70,0.80]	0.005	4
[0.0005,0.001]	0.0005	4	[0.80,0.98]	0.005	4
[0.001,0.005]	0.004	4	[0.98,0.985]	0.005	4
[0.005,0.01]	0.005	4	[0.985,0.99]	0.005	4
[0.01,0.015]	0.005	4	[0.99,0.995]	0.005	4
[0.015,0.02]	0.005	4	[0.995,0.999]	0.004	4
[0.02,0.17]	0.005	4	[0.999,0.9999]	0.0009	4
[0.17,0.30]	0.01	4	[0.9999,1.0]	0.0001	4
[0.30,0.49]	0.005	5			

Table S4. List of all simulations.

simulation #	Protein	method	time (ns)
1	WT	EQ (NaCl)	310
2	WT	EQ (KCl)	320
3	WT	US	540
4	WT	FEP	>240
5	DEAA	EQ (NaCl)	200
6	DEAA	EQ (KCl)	200
	Total simulation time: >1.61 μs		

EQ, US and FEP stand for equilibrium, umbrella sampling and free energy perturbation simulations, respectively. WT and DEAA refer to the wide-type protein and DEAA derivative, respectively.

Supplementary movies

Movie S1. Permeation of a single Na^+ ion along the identified path from extracellular bulk to the central cavity of NavPaS.

Supplementary references

Bashtannyk, D.M., and Hyndman, R.J. (2001). Bandwidth selection for kernel conditional density estimation. Computational Statistics & Data Analysis 36, 279-298.

Bennett, C.H. (1976). Efficient estimation of free energy differences from Monte Carlo data. Journal of Computational Physics 22, 245-268.

Best, R.B., Zhu, X., Shim, J., Lopes, P.E.M., Mittal, J., Feig, M., and Mackerell, A.D. (2012). Optimization of the Additive CHARMM All-Atom Protein Force Field Targeting Improved Sampling of the Backbone phi, psi and Side-Chain chi(1) and chi(2) Dihedral Angles. Journal of Chemical Theory & Computation 8, 3257-3273.

Borg, I., and Groenen, P.J. (2005). Modern multidimensional scaling: Theory and applications (Springer Science & Business Media).

Chen, R., and Chung, S.H. (2012). Binding modes of μ-conotoxin to the bacterial sodium channel (NaVAb). Biophysical Journal 102, 483-488.

Essmann, U. (1995). A smooth particle mesh Ewald method. Journal of Chemical Physics 103, 8577-8593.

Feller, S.E., Zhang, Y., Pastor, R.W., and Brooks, B.R. (1995). Constant pressure molecular dynamics simulation: The Langevin piston method. Journal of Chemical Physics 103, 4613-4621.

Frangi, A.F., Niessen, W.J., Hoogeveen, R.M., Van Walsum, T., and Viergever, M.A. (1999). Model-based quantitation of 3-D magnetic resonance angiographic images. IEEE Transactions on medical imaging 18, 946-956.

Habeck, M. (2012). Bayesian estimation of free energies from equilibrium simulations. Physical Review Letters 109, 100601.

Hub, J.S., Groot, B.L.D., and Spoel, D.V.D. (2015). g_wham—A Free Weighted Histogram Analysis Implementation Including Robust Error and Autocorrelation Estimates. Journal of Chemical Physics 6, 3713-3720.

Jones, E., Oliphant, T., and Peterson, P. (2014). SciPy: open source scientific tools for Python.

Kollman, P. (1993). Free energy calculations: Applications to chemical and biochemical

phenomena. Chemical Reviews 93, 2395.

Laptev, I., Mayer, H., Lindeberg, T., Eckstein, W., Steger, C., and Baumgartner, A. (2000). Automatic extraction of roads from aerial images based on scale space and snakes. Machine Vision and Applications 12, 23-31.

Lindeberg, T. (1998). Edge detection and ridge detection with automatic scale selection. International Journal of Computer Vision 30, 117-156.

Luo, Y., and Roux, B. (2010). Simulation of Osmotic Pressure in Concentrated Aqueous Salt Solutions. Jphyschemlett 1, 183-189.

MacKerell Jr, A.D., Bashford, D., Bellott, M., Dunbrack Jr, R.L., Evanseck, J.D., Field, M.J., Fischer, S., Gao, J., Guo, H., and Ha, S. (1998). All-atom empirical potential for molecular modeling and dynamics studies of proteins. The journal of physical chemistry B 102, 3586-3616.

Martyna, G.J., Tobias, D.J., and Klein, M.L. (1994). Constant pressure molecular dynamics algorithms. Journal of Chemical Physics 101, 4177-4189.

Miyamoto, S., and Kollman, P.A. (1992). SETTLE: an analytical version of the SHAKE and RATTLE algorithm for rigid water models. Journal of computational chemistry 13, 952-962.

Parzen, E. (1962). On estimation of a probability density function and mode. The annals of mathematical statistics 33, 1065-1076.

Pedregosa, F., Varoquaux, G., Gramfort, A., Michel, V., Thirion, B., Grisel, O., Blondel, M., Prettenhofer, P., Weiss, R., and Dubourg, V. (2011). Scikit-learn: Machine learning in Python. Journal of Machine Learning Research 12, 2825-2830.

Phillips, J.C., Braun, R., Wang, W., Gumbart, J., Tajkhorshid, E., Villa, E., Chipot, C., Skeel, R.D., Kalé, L., and Schulten, K. (2005). Scalable molecular dynamics with NAMD. Journal of Computational Chemistry 26, 1781.

Pohorille, A., Jarzynski, C., and Chipot, C. (2010). Good practices in free-energy calculations. Journal of Physical Chemistry B 114, 10235-10253.

Rosenblatt, M. (1956). Remarks on some nonparametric estimates of a density function. The Annals of Mathematical Statistics 27, 832-837.

Sato, Y., Nakajima, S., Shiraga, N., Atsumi, H., Yoshida, S., Koller, T., Gerig, G., and Kikinis, R.

(1998). Three-dimensional multi-scale line filter for segmentation and visualization of curvilinear structures in medical images. Medical image analysis 2, 143-168.

Silverman, B.W. (1986). Density estimation for statistics and data analysis, Vol 26 (CRC press).

Tenenbaum, J.B., De Silva, V., and Langford, J.C. (2000). A global geometric framework for nonlinear dimensionality reduction. science 290, 2319-2323.

Torrie, G.M., and Valleau, J.P. (1977). Nonphysical sampling distributions in Monte Carlo free-energy estimation: Umbrella sampling. Journal of Computational Physics 23, 187-199.

Tu, K., Tobias, D.J., and Klein, M.L. (1995). Constant pressure and temperature molecular dynamics simulation of a fully hydrated liquid crystal phase dipalmitoylphosphatidylcholine bilayer. Biophysical Journal 69, 2558-2562.

Turlach, B.A. (1993). Bandwidth selection in kernel density estimation: A review (Université catholique de Louvain Louvain-la-Neuve).

Zacharias, M., Straatsma, T.P., and Mccammon, J.A. (1994). Separation-shifted scaling, a new scaling method for Lennard-Jones interactions in thermodynamic integration. Journal of Chemical Physics 100, 9025-9031.

Zwanzig, R.W. (1954). High-Temperature Equation of State by a Perturbation Method. I. Nonpolar Gases. Journal of Chemical Physics 22, 1420-1426.

Example python script to identify the most probably permeation pathway from a 3D probability density map

```
# -*- coding: utf-8 -*-

import numpy as np
from numpy.linalg import inv, eigh, norm

from scipy import ndimage, stats
from scipy.linalg import eigh as scipyeigh
from scipy.sparse.csgraph import floyd_warshall

from sklearn.manifold import Isomap

# Read Positions
FileName = 'Test_position.txt'
cors = np.loadtxt(FileName)
# Determine the boundary of the positions
minc, maxc = cors.min(axis=0), cors.max(axis=0)
minc = np.floor(minc)-1
maxc = np.ceil(maxc)+1

# Use 0.5A as grid step which could be modified.
step = 0.5
# Use KDE to build the density
kernel = stats.gaussian_kde(cors.T, bw_method='silverman')
X, Y, Z = np.mgrid[minc[0]:maxc[0]:step, minc[1]:maxc[1]:step, minc[2]:maxc[2]:step]
positions = np.vstack([X.ravel(), Y.ravel(), Z.ravel()])
density = np.reshape(kernel(positions), X.shape)
gridpos = [np.arange(minc[i], maxc[i]+step, step) for i in range(3)]
# The local region size to approximate the derivative
regsize = 3
ind = np.indices((regsize*2+1, regsize*2+1, regsize*2+1))-regsize
ind = ind.reshape((3, -1))
tempinfo = np.vstack((np.ones(ind.shape[1]), ind, ind**2*.5, ind[0]*ind[1], ind[0]*ind[2],
ind[1]*ind[2])).T
tempinfo = ((inv(tempinfo.T.dot(tempinfo)).dot(tempinfo.T)))
tempinfo.resize(10, regsize*2+1, regsize*2+1, regsize*2+1)
tempinfo = tempinfo[:, ::-1, ::-1, ::-1]
# Use a series of scale
sigmas = np.arange(1, 20, 1)
infos = np.zeros((len(sigmas), density.shape[0], density.shape[1], density.shape[2], 10))
```

```
dd = np.zeros([len(sigmas)]+list(density.shape)+[3])
dd[:] = 999
strength = np.zeros([len(sigmas)]+list(density.shape))
for i, s in enumerate(sigmas):
    tempmatrix = ndimage.gaussian_filter(density, s, mode='mirror')
    for j in range(10):
        infos[i, :, :, :, j] = ndimage.convolve(tempmatrix, tempinfo[j], mode='nearest')
    hessian = infos[i][:, :, :, [[4, 7, 8], [7, 5, 9], [8, 9, 6]]]
    grad = infos[i][:, :, :, [1, 2, 3]]
    e, v = eigh(hessian)
    A = np.stack(((e[:, :, :, np.newaxis, 0]*v[:, :, :, :, 0]), (e[:, :, :, np.newaxis, 1]*v[:, :, :, :, 1]),
v[:, :, :, :, 2]), 3)
    b = -np.stack((((v[:, :, :, :, 0]*grad[:, :, :]).sum(3), (v[:, :, :, :, 1] * grad[:, :, :]).sum(3),
(v[:, :, :, :, 2]*grad[:, :, :]).sum(3)*0), 3)
    for ii in range(density.shape[0]):
        for jj in range(density.shape[1]):
            for kk in range(density.shape[2]):
                try:
                    dd[i, ii, jj, kk] = (inv(A[ii, jj, kk]).dot(b[ii, jj, kk][:, np.newaxis]))[:, 0]
                except:
                    pass
    mask1 = (e[:, :, :, :2] < 0).all(axis=3)
    mask2 = norm(dd[i], axis=3) < 3**.5
    mask = mask1*mask2
    strength[i, :, :, :] = (e[:, :, :, 0]**2+e[:, :, :, 1]**2)*s**4.
    strength[i, mask == False] = 0
argm = strength.argmax(axis=0)
xind, yind, zind = np.indices(density.shape)
gridposes = np.array((xind, yind, zind))
topoint = np.transpose(gridposes, (1, 2, 3, 0))+dd[argm, xind, yind, zind]
width = sigmas[argm]*step
width = width[(strength[argm, xind, yind, zind] != 0)*(density > 0.001)]
topoint = topoint[(strength[argm, xind, yind, zind] != 0)*(density > 0.001)]
width = width[(topoint > 0).all(axis=1) * (topoint < np.array(density.shape)-1).all(axis=1)]
topoint = topoint[(topoint > 0).all(axis=1) * (topoint < np.array(density.shape)-1).all(axis=1)]
findridge = topoint*step+minc
# Futher optimize the ridge points
for ccc in range(10):
    dd = np.zeros([len(sigmas), len(topoint), 3])
    dd[:] = 999
    strength = np.zeros([len(sigmas), len(topoint)])
    indexint = np.floor(topoint).astype(int)
```

```
    indexfloat = topoint-indexint
    infohere = ( infos[:, indexint[:, 0], indexint[:, 1], indexint[:, 2]]*((1-indexfloat[:, 0])*(1-indexfloat[:, 1])*(1-indexfloat[:, 2]))[np.newaxis, :, np.newaxis] +
                 infos[:, indexint[:, 0]+1, indexint[:, 1], indexint[:, 2]]*((indexfloat[:, 0])*(1-indexfloat[:, 1])*(1-indexfloat[:, 2]))[np.newaxis, :, np.newaxis] +
                 infos[:, indexint[:, 0], indexint[:, 1]+1, indexint[:, 2]]*((1-indexfloat[:, 0])*(indexfloat[:, 1])*(1-indexfloat[:, 2]))[np.newaxis, :, np.newaxis] +
                 infos[:, indexint[:, 0], indexint[:, 1], indexint[:, 2]+1]*((1-indexfloat[:, 0])*(1-indexfloat[:, 1])*(1-indexfloat[:, 2]))[np.newaxis, :, np.newaxis] +
                 infos[:, indexint[:, 0]+1, indexint[:, 1]+1, indexint[:, 2]]*((indexfloat[:, 0])*(indexfloat[:, 1])*(indexfloat[:, 2]))[np.newaxis, :, np.newaxis] +
                 infos[:, indexint[:, 0]+1, indexint[:, 1], indexint[:, 2]+1]*((indexfloat[:, 0])*(1-indexfloat[:, 1])*(indexfloat[:, 2]))[np.newaxis, :, np.newaxis] +
                 infos[:, indexint[:, 0], indexint[:, 1]+1, indexint[:, 2]+1]*((1-indexfloat[:, 0])*(indexfloat[:, 1])*(indexfloat[:, 2]))[np.newaxis, :, np.newaxis] +
                 infos[:, indexint[:, 0]+1, indexint[:, 1]+1, indexint[:, 2]+1]*((indexfloat[:, 0])*(indexfloat[:, 1])*(indexfloat[:, 2]))[np.newaxis, :, np.newaxis])
    for i, s in enumerate(sigmas):
        hessian = infohere[i][:, [[4, 7, 8], [7, 5, 9], [8, 9, 6]]]
        grad = infohere[i][:, [1, 2, 3]]
        e, v = eigh(hessian)
        A = np.stack(((e[:, np.newaxis, 0]*v[:, :, 0]), (e[:, np.newaxis, 1]*v[:, :, 1]), v[:, :, 2]), 1)
        b = -np.stack(((v[:, :, 0]*grad[:]).sum(1), (v[:, :, 1] * grad[:]).sum(1), (v[:, :, 2]*grad[:]).sum(1)*0), 1)
        for ii in range(len(topoint)):
            try:
                dd[i, ii] = (inv(A[ii]).dot(b[ii][:, np.newaxis]))[:, 0]
            except:
                pass
        mask1 = (e[:, :2] < 0).all(axis=1)
        mask2 = norm(dd[i], axis=1) < 3**.5
        mask = mask1*mask2
        strength[i, :] = (e[:, 0]**2+e[:, 1]**2)*s**4.
        strength[i, mask == False] = 0
    argm = strength.argmax(axis=0)
    topoint = topoint+dd[argm, np.arange(len(topoint))]
    width = sigmas[argm]*step
    width = width[strength[argm, np.arange(len(topoint))] != 0]
    topoint = topoint[strength[argm, np.arange(len(topoint))] != 0]
    width = width[(topoint > 0).all(axis=1) * (topoint < np.array(density.shape)-1).all(axis=1)]
    topoint = topoint[(topoint > 0).all(axis=1) * (topoint < np.array(density.shape)-1).all(axis=1)]
```

```
findridge = topoint*step+minc
# Isomap
isomap=Isomap(n_neighbors=400,n_jobs=8,n_components=2)
isomap.fit(findridge)
distmap=isomap.dist_matrix_
distmap[distmap==0]=np.inf
shortest=floyd_warshall(distmap)**2
l1=shortest.mean(0)[:,np.newaxis]
l2=shortest.mean(0)[np.newaxis,:]
b=-.5*(shortest-l1-l2+shortest.mean())
e,v=scipyeigh(b,eigvals=(len(findridge)-10,len(findridge)-1))
print('\n'.join(["Dimension {}: {:.2f}%".format(i+1,e[-i-1]**2/(e**2).sum()*100) for i in
range(10)]))
sortedupcor=findridge[np.argsort(v[:,-1])]
findpos=[]
for i in np.linspace(v[:,-1].min(),v[:,-1].max(),200):
    temppos=np.zeros((1,len(e)))
    temppos[:,-1]=i
    weight=np.exp(-norm((v-temppos)*e,axis=1)*.01)
    weight/=weight.sum()
    findpos.append((weight[:,np.newaxis]*findridge).sum(axis=0))
findpos=np.array(findpos)
np.savetxt('Result.txt',findpos,fmt='%.3f')
```
